# Highly pathogenic avian influenza virus infection in chickens but not ducks is associated with elevated host immune and pro-inflammatory responses

**DOI:** 10.1186/s13567-014-0118-3

**Published:** 2014-11-28

**Authors:** Suresh V Kuchipudi, Meenu Tellabati, Sujith Sebastian, Brandon Z Londt, Christine Jansen, Lonneke Vervelde, Sharon M Brookes, Ian H Brown, Stephen P Dunham, Kin-Chow Chang

**Affiliations:** School of Veterinary Medicine and Science, University of Nottingham, Sutton Bonington Campus, College Road, Loughborough, Nottingham, Leicestershire LE12 5RD UK; Virology Department, Animal and Plant Health Agency, Weybridge, Addlestone, Surrey, KT15 3NB UK; Department of Infectious Diseases and Immunology, Faculty of Veterinary Medicine, University of Utrecht, Utrecht, The Netherlands; Current address: The Roslin Institute and R(D)SVS, University of Edinburgh, Easter Bush, Midlothian, Edinburgh, EH25 9RG UK

## Abstract

Highly pathogenic avian influenza (HPAI) H5N1 viruses cause severe infection in chickens at near complete mortality, but corresponding infection in ducks is typically mild or asymptomatic. To understand the underlying molecular differences in host response, primary chicken and duck lung cells, infected with two HPAI H5N1 viruses and a low pathogenicity avian influenza (LPAI) H2N3 virus, were subjected to RNA expression profiling. Chicken cells but not duck cells showed highly elevated immune and pro-inflammatory responses following HPAI virus infection. HPAI H5N1 virus challenge studies in chickens and ducks corroborated the in vitro findings. To try to determine the underlying mechanisms, we investigated the role of signal transducer and activator of transcription-3 (STAT-3) in mediating pro-inflammatory response to HPAIV infection in chicken and duck cells. We found that *STAT-3* expression was down-regulated in chickens but was up-regulated or unaffected in ducks in vitro and in vivo following H5N1 virus infection. Low basal STAT-3 expression in chicken cells was completely inhibited by H5N1 virus infection. By contrast, constitutively active STAT-3 detected in duck cells was unaffected by H5N1 virus infection. Transient constitutively-active STAT-3 transfection in chicken cells significantly reduced pro-inflammatory response to H5N1 virus infection; on the other hand, chemical inhibition of STAT-3 activation in duck cells increased pro-inflammatory gene expression following H5N1 virus infection. Collectively, we propose that elevated pro-inflammatory response in chickens is a major pathogenicity factor of HPAI H5N1 virus infection, mediated in part by the inhibition of STAT-3.

**Electronic supplementary material:**

The online version of this article (doi:10.1186/s13567-014-0118-3) contains supplementary material, which is available to authorized users.

## Introduction

Avian influenza A viruses continue to spread globally causing millions of poultry deaths and are significant zoonotic pathogens [1]. In particular, Eurasian lineage highly pathogenic avian influenza (HPAI) H5N1 virus infection causes severe disease in humans with a fatality rate of about 60% [2]. Most human influenza pandemics of the 20^th^ century had been caused by influenza A viruses (IAVs) that originated, either wholly or in part, from avian influenza A viruses [3]. Ducks and waterfowl are reservoirs for most IAVs, including the hemagglutinin (HA) and neuraminidase (NA) subtypes that have caused previous human pandemics [4]. Despite being susceptible to infection with a wide range of IAVs, such birds often show little or no clinical signs [5,6].

In contrast, most HPAI H5N1 virus strains produce very severe disease in chickens, turkeys and quails often causing up to 100% mortality within 2–3 days [7,8]. With their natural resistance, ducks support genetic reassortment of influenza viruses providing a mechanism of evolution of genetically diverse IAVs including HPAI H5N1 viruses [9-11]. The rapid onset of fatal disease in chickens and no evidence of clinical disease in ducks suggests that there are potential differences in the innate immune mechanisms between these two important avian hosts. Recent evidence shows that the resistance of ducks to HPAI virus infection is not absolute. Contemporary Eurasian lineage HPAI H5N1 viruses have caused large numbers of deaths in both poultry and water fowl including ducks. Experimental infection of Pekin ducks (*Anas platyrhynchos*) with a HPAI H5N1 clade 2.2.1 virus (A/turkey/Turkey/1/2005) causes fatal infection [12], suggesting that certain clades of contemporary Eurasian lineage HPAI H5N1 viruses are able to overcome the natural innate resistance of ducks.

The unusual severity of HPAI H5N1 virus infection in humans, in contrast to seasonal H3N2 or H1N1 influenza viruses, has been regarded to be due to hyper-acute induction of pro-inflammatory cytokines often referred as hypercytokinemia or cytokine storm [13-15]. Pigs show mild or no clinical signs following HPAI H5N1 virus infection [16]. We recently showed that the innate resistance of pigs to HPAI H5N1 virus is mediated through reduced pro-inflammation and infectious virus release [17]. These findings indicate that dysregulation of host pro-inflammatory response to infection is a key contributing factor to the morbidity and mortality of virulent influenza virus infections. Similarly a recent study found that excessive delayed inflammatory cytokine responses may contribute to the severe pathogenicity of HPAI H7N1 in chickens [18]. However, the pathophysiology of H5N1 virus infection in chickens and ducks remains unclear. To further our molecular understanding of the pathogenesis of HPAI H5N1 virus infection in chickens and ducks, we examined differences in host gene response to IAV infection between chickens and ducks in vitro (in lung cells) and in vivo.

## Materials and methods

### Viruses

A low pathogenicity avian influenza virus (A/mallard duck/England/7277/06, referred to as LPAI-H2N3), a classical HPAI H5N1 virus strain (A/turkey/England/50-92/91, referred to as H5N1-tyEng91) and a contemporary Eurasian lineage clade 2.2.1 HPAI H5N1 virus (A/turkey/Turkey/1/05, referred to as H5N1-tyTR05) were used in this study. While the “classical” H5N1 virus typically causes non-lethal infection in ducks [19], the contemporary Eurasian lineage (clade 2.2.1) H5N1 virus may cause severe disease with mortality in ducks [12]. All viruses were grown in 10-day-old embryonated chicken eggs by allantoic inoculation.

### Primary cells and virus infection

Primary cell cultures were isolated from lungs of 4-week-old broiler chickens and 4-week-old Pekin ducks as previously described [20]. Cells were grown in collagen coated cell culture flasks (Costar, Corning, UK) in Dulbecco’s Modified Eagle’s Medium (DMEM) and Ham’s F12 (1:1) supplemented with 2% chicken embryo extract (Biosera, Uckfield, UK), 5% fetal bovine serum, 1% insulin-transferrin selenium (Life Technologies, Paisley, UK) and antibiotics. Monolayers of primary cells in 6 well cell culture plates (Costar) were infected with LPAI or HPAI viruses at multiplicity of infection (MOI) of 1.0. Three wells of avian cells were used for each virus infection. Mock infections were performed without virus in triplicate wells for each cell type. Cells were rinsed with phosphate buffered saline (PBS) and infected with appropriate amount of the virus in serum free infection medium comprising 2% Ultroser G (Pal Biosepra, Cedex, France), 500 ng/mL TPCK trypsin (Sigma-Aldrich, Dorset, UK) and antibiotics in Ham’s F12 medium. After 2 h incubation with the virus, the cells were washed three times with PBS and fresh medium was added.

### Immuno-staining for virus nucleoprotein (NP)

To determine the pattern of virus infection, virus and mock infected cells were fixed in acetone:methanol at 6 hours post-infection (hpi) and were subjected to viral nucleoprotein detection by a primary mouse monoclonal antibody (Abcam, Cambridge, UK) followed by visualization with Envision + system-HRP (DAB; Dako, Ely, UK). Cell culture supernatants form infected cells were titrated in MDCK cells to determine focus forming units (ffu) using an immuno-cytochemical focus assay as previously described [20].

### Microarray gene expression profiling

At 24 hpi, total RNA from each well was extracted using RNeasy Plus Mini - QIAshredder Kit (Qiagen, Manchester, UK) and the quality of the total RNA samples was determined using a RNA 6000 nano kit (Agilent 2100 Bioanalyzer, Agilent Technologies, Stockport, UK) following the manufacturer’s instructions. Microarray expression analysis was carried out using GeneChip chicken genome arrays (Affymetrix, High Wycombe, UK). Duplicate RNA samples from each of virus or mock infected chicken and duck cells were used for microarray analysis and a total of 16 array chips (3 viruses × 2 avian species × duplicate, plus 2 chicken and 2 duck mock infected) were used in the study.

Microarray expression data were analyzed using GeneSpring GX11 expression analysis software (Agilent Technologies) [21,22]. Functional clustering of data was carried out using DAVID bioinformatics resources version 6.7 [23,24].

To take into account the specificity of heterologous hybridization (between labelled duck targets on chicken probes), a well-established analytical tool was used which involved the hybridization of duck genomic DNA to the chicken chip to establish specific probe binding for duck transcriptome analysis using chicken GeneChip arrays.

Cross species array analysis was performed by generating a probe masking file to select probe-sets on the chicken chip for subsequent duck transcriptome analyses if the probe-set was represented by perfect match (PM) probes with duck gDNA hybridization intensities above an experimentally set threshold [25-27]. An additional file shows the detailed protocols of microarray expression study including the cross species hybridization data analysis (see Additional file 1).

### Quantitative reverse transcription PCR (qRT-PCR) for viral and host genes

Viral RNA was extracted from culture media using QIAamp Viral RNA Mini Kit (Qiagen). One-step qRT-PCR to quantify influenza viral matrix gene was performed as previously described [20]. Based on the comparison of global gene expression profiles of chicken and duck cells, key pro-inflammatory and antiviral genes were selected and validated by qRT-PCR using the same total RNA samples as that used for the microarray experiment.

Oligonucleotide primers and hydrolysis probes for TaqMan assays were designed from published sequences using Primer Express software version 3.0.1 (Applied Biosystems, Life Technologies). All primers were provided by Eurofins Genomics (Edersberg, Germany) and all probes were supplied by Sigma Aldrich. Primer and probe sequences are shown in Table 1. qRT-PCR assays for lipopolysaccharide induced TNF alpha factor (*LITAF*) and *STAT-3* genes were performed using SYBR green method using same set of primers for both chicken and duck. Melting curve analysis was performed to ensure the specificity of the SYBR green PCR. qRT-PCR of cDNA samples converted from total RNA (Superscript III First-strand cDNA synthesis system, Life Technologies) was performed on a the *LightCycler*® 480 (Roche, Burges Hill, UK), and using a relative standard curve method normalized *to 18S* ribosomal RNA (18SrRNA) expression.

**Table 1:** **Primer and probe sequences for quantitative reverse transcription PCR assays**

**Gene**	**GenBank acc. No**	**Primer sequence**	**Probe sequence**
**Chicken**			
18S rRNA	AF173612.1	Fwd :TGTGCCGCTAGAGGTGAAATT	5′ (6FAM) TTGGACCGGCGCAAGACGAAC 3′ (TAMRA)
		Rev: TGGCAAATGCTTTCGCTTT	
IL-6	EU170468	Fwd :CACGATCCGGCAGATGGT	5′ (6FAM)ATAAATCCCGATGAAGTGGTCATCC 3′ (TAMRA)
		Rev: TGGGCGGCCGAGTCT	
IL-8 /CXCLi1(K60)	NM_205018.1	Fwd :CCCTCGCCACAGAACCAA	5′ (6FAM)CCCAGGTGACACCCGGAAGAAACA 3′ (TAMRA)
		Rev: CAGCCTTGCCCATCATCTTT	
IFN-α	EU367971	Fwd :CTTCCTCCAAGACAACGATTACAG	5′ (6FAM)CCTGCGCCTGGGAACACGTCC 3′ (TAMRA)
		Rev: AGGAACCAGGCACGAGCTT	
LITAF	AY765397	Fwd :CCCTTCTGAGGCATTTGGAA	
		Rev: CAGCCTGCAAATTTTGTCTTCTT	
STAT-3	NM_001030931.1	Fwd: TGGGTGGAGAAGGACATCA	
		Rev: CATGGGCAGGTCAATGGTAT	
Duck			
Duck IL-6	AB191038	Fwd :CCAAGGTGACGGAGGAAGAC	5′ (6FAM)TGTCTCCTGGCTGGCTTCGACGA 3′ (TAMRA)
		Rev: TGGAGAGTTTCTTCAAGCATTTCTC	
Duck IL-8	AB236334.1	Fwd :AGCCTGGTAAGGATGGGAAAC	5′ (6FAM)AGCTCCGGTGCCAGTGCATAAGCA 3′ (TAMRA)
		Rev: GGGTGGATGAACTTCGAGTGA	
Duck IFN-α	DQ861429	Fwd :AACCAGCTTCAGCACCACATC	5′ (6FAM)TGCTTCCCAGCCGACGCC 3′ (TAMRA)
		Rev: TGTGGTTCTGGAGGAAGTGTTG	

### HPAI H5N1 virus challenge in chickens and ducks

Three-week-old Lohmann Brown chickens kept in containment level 3 facilities (AHVLA, Weybridge) were infected with HPAI H5N1-tyTR05 virus. Chickens were inoculated intranasally and intraocularly with 0.1 mL of 1 × 10^6^ EID_50_ virus diluted in PBS. Birds were killed at 24 h after infection (three birds each from virus and control groups), lung and spleen tissues were collected and stored at −80 °C prior to RNA extraction. Three- weeks- old Pekin ducks were inoculated with 0.1 mL of 1 × 10^6^ EID_50_ of H5N1-tyTR05 virus intranasally and intraocularly. Birds were killed humanely at 24 hpi (three birds each from virus and control groups), lung and spleen tissues were collected and stored at −80 °C prior to RNA extraction. Tissues were homogenized using GentleMacs Dissociator (Miltenyi Biotec, Bisley, UK) and total RNA was extracted from the homogenized tissues using RNeasy Mini-Kit (Qiagen) following the manufacturer’s instructions. HPAI H5N1 virus challenge studies were performed with AHVLA committee ethical approval and in accordance with the UK 1986 Animal Scientific Procedure Act and AHVLA code of practice for performance of scientific studies using animals [License number 70/7062].

### STAT-3 over-expression and chemical inhibition

Based on the high sequence identity of STAT-3 protein (97%) between chicken and mouse, we used a mouse STAT-3 expression plasmid for this study. Primary chicken embryo cells in 6-well culture plates (Costar) were transiently transfected with constitutively active mouse STAT-3 expression plasmid [Stat3-C Flag pRc/CM V, plasmid 8722, Addgene, USA] or empty pRc/CMV vector (Invitrogen) using TransIT-LT1 reagent (Mirus Bio, Cambridge, UK). At 80% cell confluence, transfection mixture containing 250 μL Optimem, 2.5 μg plasmid DNA and 7.5 μL of TransIT reagent were added. Three days post-transfection, cells were infected with H5N1-tyEng91 virus at MOI of 1.0.

Primary duck embryo cells were treated with STAT-3 inhibitor S3I-201 (Calbiochem, Merck, Nottingham, UK), a cell-permeable amidosalicylic acid compound that binds STAT3-SH2 domain and prevents STAT3 phosphorylation/activation, dimerization and DNA-binding, at a final concentration of 100 μM [28] or vehicle (DMSO) control, one day before infection. Duck cells were pre-incubated with H5N1- tyEng91 virus at MOI of 1.0 for 2 h without S3I-201, after two hours the medium was removed, cells were rinsed with PBS and fresh medium with S3I-201 was replaced. At 24 hpi, cell lysates were harvested for protein and total RNA extractions. Western blotting was performed to detect phospho-STAT-3 (#9131, Cell Signaling Technology, Hitchin, UK) and influenza nucleoprotein (NP) (AA5H, #ab 20343, Abcam).

### Statistical analysis

qRT-PCR data was subjected to statistical analysis by a randomization test with a pair-wise reallocation using relative expression analysis software tool (REST^©^) [29].

## Results

### Comparable susceptibility to influenza virus infection and viral RNA accumulation in chicken and duck cells

**Figure 1:**
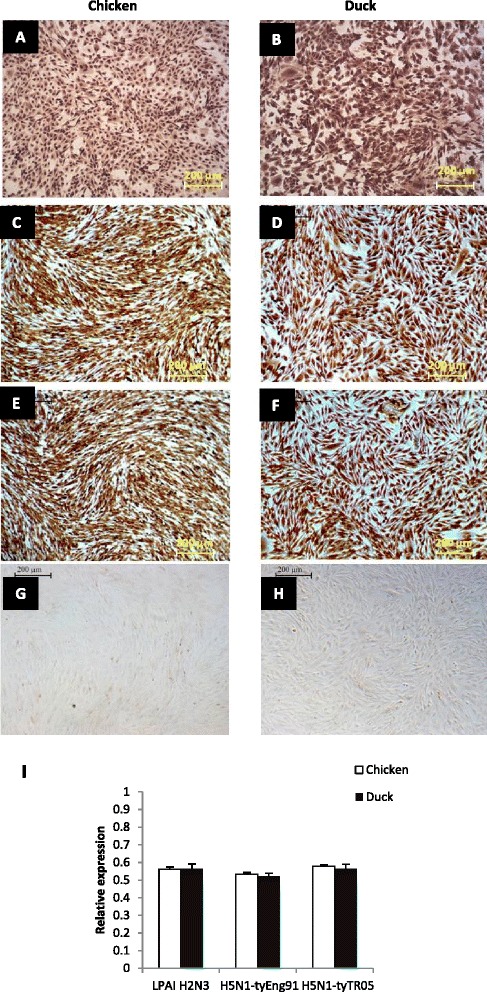
**Chicken and duck cells showed comparable susceptibility to influenza virus infection and viral RNA accumulation.** At 6 hpi with LPAI H2N3, H5N1 tyEng91 or H5N1 tyTR05 virus infection at MOI 1.0, similar accumulation of influenza nucleoprotein (NP) was evident in chicken **(A,** **C,** **E)** and duck **(B,** **D,** **F)** cells as detected by immunocytochemistry. Mock-infected chicken **(G)** and duck **(H)** cells show no staining. Comparable accumulation of viral matrix gene RNA between chicken and duck cells at 24 hpi at 1.0 MOI with LPAI H2N3, H5N1-tyEng91 or H5N1 tyTR05 viruses **(I)**. Data were derived from biological replicates of 3 total RNA samples and the data points are mean relative expression values normalized to 18SrRNA expression.

Infection of primary chicken and duck lung cells with LPAI-H2N3, H5N1-tyEng91 or H5N1-tyTR05 at 1.0 multiplicity of infection (MOI) based on MDCK cell titration resulted in comparable levels of virus infection as determined by virus NP detection by immunocytochemistry at 6 hpi (Figure 1A to H). Influenza virus matrix gene expression at 24 hpi with LPAI-H2N3, H5N1-tyEng91 and H5N1-tyTR05 viruses was comparable in chicken and duck cells (Figure 1).

### More immune-related genes in chicken cells than duck cells were induced by influenza virus infection

**Figure 2:**
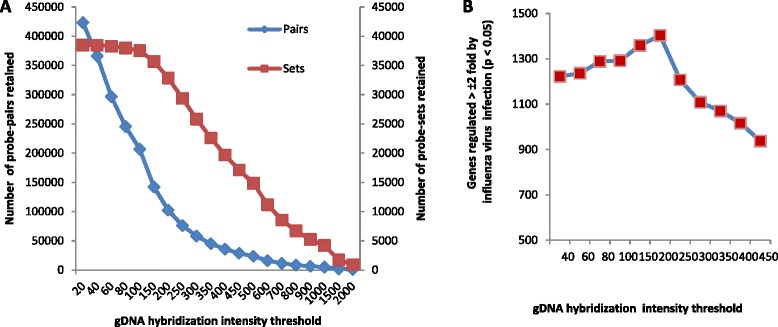
**Genomic DNA (gDNA) hybridization intensity threshold of 200 provided the highest sensitivity for duck transcriptomic analysis on the chicken GeneChip. (A)** The retention of whole probe-sets from duck gDNA hybridization on the chicken GeneChip array, representing transcripts, was less sensitive to the increase in gDNA hybridization intensities as only a minimum of one probe pair is required to retain a probe-set. **(B)** gDNA hybridization intensity threshold of 200 gave the highest number of significantly differentially regulated genes at ±2 fold (*p* ≤0.05) at 24 h following influenza virus infection compared with mock-infected controls. Data derived from hybridizing infected and mock-infected control duck RNA samples on chicken array.

A DNA microarray based global gene expression approach with a chicken GeneChip array (Affymetrix) was used to identify differences in gene expression between chicken and duck primary lung cells in response to 24 h of infection with LPAI H2N3, H5N1-tyEng91 or H5N1-tyTR05 viruses. Microarray datasets are available on the gene expression omnibus (GEO) site under accession number GSE33389 [30]. “Probe mask file” generated with a duck genomic DNA hybridization intensity threshold of 200 provided the highest sensitivity for duck expression analysis on the chicken GeneChip platform (Figure 2). Probe masking resulted in a loss of 5639 transcripts out of the total 38 535 transcripts represented in the original chicken GeneChip technology.

With one-way ANOVA and filtering at a *p* < 0.05 from hybridization results of all 3 virus subtypes, 18 783 out of 38 535 transcripts (48.74%) in chicken cells and, but only 7686 out of 32 896 transcripts (23.36%) in duck cells, were differentially regulated relative to corresponding controls.

**Figure 3:**
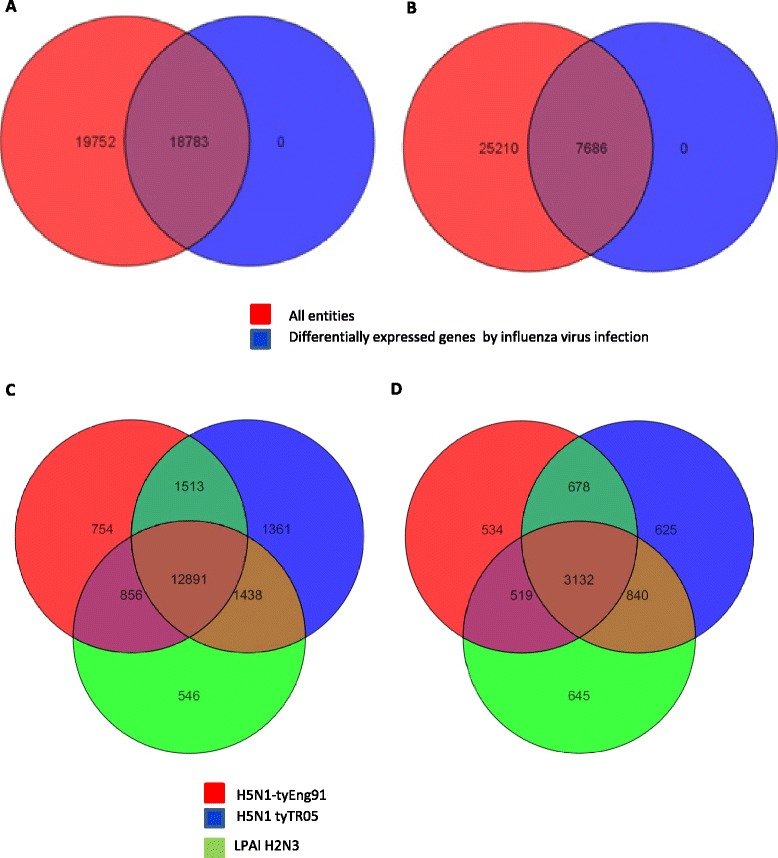
**Summary of global gene expression in chicken and duck cells in response to influenza virus infection.** Combined gene expression profiles of virus infected (all three avian viruses combined) and mock-infected samples showed that 18 783 out of 38 535 transcripts (48.74%) were significantly differentially regulated (*P* < 0.05) in chicken cells **(A)** while only 7686 out of 32 896 transcripts (23.36%) were significantly differentially regulated (*P* < 0.05) in duck cells **(B)** at 24 h following virus infection. Venn diagram overlap of significantly differentially regulated genes with a fold change of ±1.3 (≥ 1.3 fold, *p* ≤ 0.05) in **(C)** chicken cells and **(D)** duck cells at 24 h following infection with H5N1-tyEng91 (red), H5N1 tyTR05 (blue) or LPAI H2N3 (green) viruses.

The overlap among filtered genes based on fold change difference of virus infected (all three viruses) against mock infected samples provided a quantitative view of genes that were differentially expressed in chicken (Figure 3A) and duck (Figure 3B) cells following infection. Of the total number of differentially expressed genes, 12 891 genes (33.45%) in chicken cells and 3132 genes (9.52%) in duck cells were common to all three viruses. Further comparative analysis showed that 546, 754 and 1361 genes were unique to LPAI H2N3, H5N1-tyEng91 and H5N1-tyTR05 infected chicken cells respectively (Figure 3C). In duck cells, 645, 534 and 625 genes were unique to LPAI H2N3, H5N1-tyEng91 and H5N1-tyTR05 infection respectively (Figure 3D). Gene expression profiles were further analysed using the functional annotation tool in DAVID bioinformatics resources 6.7. Genes representing cytokines, chemokines, members of immunoglobulin super family, major histocompatibility complex (MHC), genes involved in T and B lymphocyte function and components of immune signalling pathways such as toll like receptor (TLR) pathway, and janus kinase (JAK) - signal transducer and activator of transcription (STAT) (JAK-STAT) pathway were classified as “immune” genes.

Several genes involved in key biological functions such as enzymes and transcription factors were down-regulated in HPAI H5N1virus infected chicken cells while most of these genes were either up-regulated or not affected in HPAI H5N1 virus infected duck cells (Table 2).

**Table 2:** **Differential expression of genes involved in key biological functions between HPAIV infected chicken and duck cells (detected by microarray)**

**Gene symbol**	**Gene name**	**Entrez Gene ID**	**Chicken cells**				**Duck cells**			
			**H5N1-tyEng91**		**H5N1-tyTR05**		**H5N1-tyEng91**		**H5N1-tyTR05**	
			**Fold change**	**Regulation**	**Fold change**	**Regulation**	**Fold change**	**Regulation**	**Fold change**	**Regulation**
**Signal transduction**										
PRKAR2A	Protein kinase, cAMP-dependent, regulatory, type II, alpha	416062	2.27	up	4.59	down	3.96	down	5.86	down
IPO7	Importin 7	423046	-	unchanged	2.21	down	4.83	up	2.99	down
**GTPase inhibitor activity**										
GPS1	G protein pathway suppressor 1	417382	2.86	down	3.01	down	2.21	up	3.2	up
Lipid metabolism, production of ROS										
ACOX1	Acyl-Coenzyme A oxidase 1, palmitoyl	417366	4.33	down	2.88	down	-	unchanged	3.24	up
**Enzymes**										
B4GALNT3	Beta-1,4-N-acetyl-galactosaminyl transferase 3	418150	2.07	down	1.33	up	2.58	up	2.08	up
DNPEP	Aspartyl aminopeptidase	424200	2.38	up	1.3	up	-	unchanged	1.44	down
**Catalytic activity (vit B6 metabolism)**										
PSAT1	Phosphoserine aminotransferase 1	427263	4.38	down	3.9	down	11.44	up	11.69	up
**Transcription factor**										
RREB1	Ras responsive element binding protein 1	395920	3.41	down	2.26	down	2.73	up	7.31	up
**Isoleucyl-tRNA aminoacylation**										
IARS2	Isoleucyl-tRNA synthetase 2, mitochondrial	421346	17.58	down	22.92	down	14.52	up	38.82	up
**Peptidolysis, IL-4 biosynthesis**										
MMP28	Matrix metallopeptidase 28	417523	2.66	down	1.93	down	3.31	up	10.38	up

Many more immune-related genes were differentially regulated in chicken cells than in ducks cells in response to infection with the two HPAI H5N1 viruses. Of the 75 immune-related genes that were significantly up-regulated in HPAI H5N1-tyEng91 virus infected chicken cells (fold change ≥ 1.3 and *p* < 0.05), expression of 63 genes (84%) was not significantly affected (*p* > 0.05) and 12 genes (16%) were significantly down-regulated (*p* < 0.05) in corresponding duck cells. Full list of immune related genes that were differentially regulated between H5N1 virus infected chicken and duck cells is provided as an additional file (see Additional file 2). A similar difference in immune related gene expression was also observed between chicken and duck cells at 24 h H5N1-tyTR05 virus infection (see Additional file 2). However, some of these immune genes were down-regulated in chicken cells infected with LPAI H2N3 and the genes that were up-regulated showed a lower fold increase than those in the HPAI infected chicken cells (see Additional file 2).

### Pro-inflammatory genes were up-regulated in infected chickens (lung cells and in vivo) but not in ducks

Pro-inflammatory cytokine genes, *interleukin* (*IL*)- *6*, *IL-8* (CXCLi1) and *IL-10*, were highly up-regulated in both HPAI H5N1 virus infected chicken cells; in contrast, *IL-8* expression was unchanged, and *IL-6* and *IL-10* were down regulated in infected duck cells with the same viruses (Table 3). Expression of *IL-18* was up-regulated in duck cells but was down-regulated in chicken cells following infection with H5N1-tyEng91 or H5N1-tyTR05 viruses (Table 3).

**Table 3:** **Differential expression of key immune related genes between HPAIV infected chicken and duck cells (detected by microarray)**

**Gene symbol**	**Gene name**	**Entrez Gene ID**	**Chicken cells**				**Duck cells**			
			**H5N1-tyEng91**		**H5N1-tyTR05**		**H5N1-tyEng91**		**H5N1-tyTR05**	
			**Fold change**	**Regulation**	**Fold change**	**Regulation**	**Fold change**	**Regulation**	**Fold change**	**Regulation**
**JAK-STAT Pathway**										
STAT3	signal transducer and activator of transcription 3	420027	2.33	down	2.72	down	1.39	up	-	unchanged
JAK1	Janus kinase 1 (a protein tyrosine kinase)	554219	2.79	down	3.21	down	-	Removed*	-	Removed*
IFNAR1	Interferon (alpha, beta and omega) receptor 1	395665	8.02	down	16.65	down	13.91	up	1.37	up
PIAS2	Protein inhibitor of activated STAT, 2	416383	5.05	down	3.86	down	1.62	up	3.7	down
**Cytokines and Chemokines**										
IL8/ CXCLi1(K60)	interleukin 8	395872	232.8	up	2.96	up	-	unchanged	-	unchanged
IL6	interleukin 6 (interferon, beta 2)	395337	131.08	up	10.66	up	2.92	down	-	unchanged
IL10	interleukin 10	428264	1.39	up	1.6	up	1.39	down	-	unchanged
IL18	interleukin 18 (interferon-gamma-inducing factor)	395312	4.7	down	4.14	down	3.02	up	2.4	up

*Transcript removed during probe masking.

**Figure 4:**
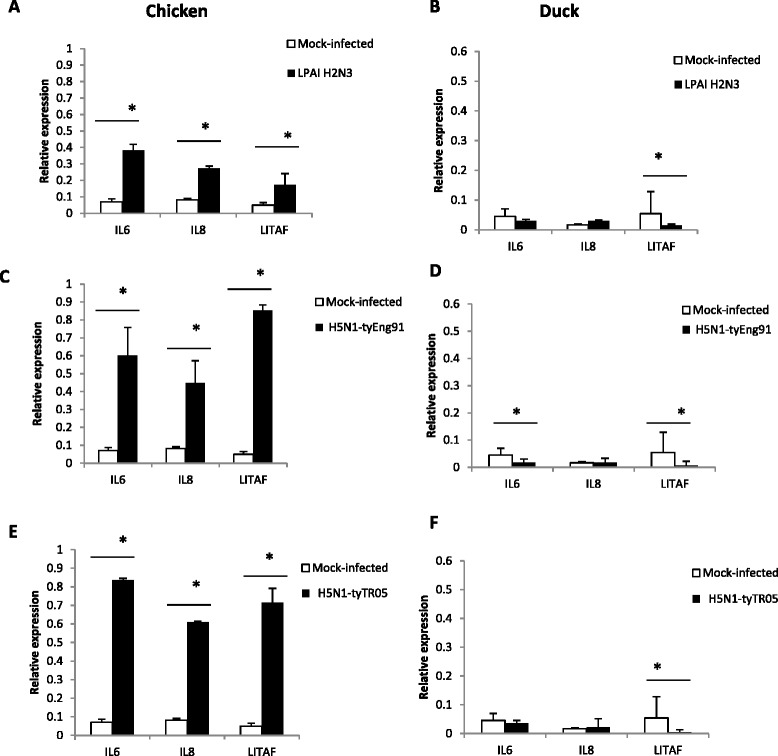
**Contrasting pro-inflammatory cytokine gene response between chicken and duck cells.** In chicken cells at 24 h following infection with **(A)** LPAI H2N3, **(C)** H5N1-tyEng91 or **(E)** H5N1-tyTR05 viruses, mRNA expression levels of *IL-6*, *IL-8* and *LITAF* were significantly up-regulated. In duck cells at 24 h following infection with **(B)** LPAI H2N3 **(D)** H5N1-tyEng91 or **(F)** H5N1-tyTR05 viruses, *IL-6*, *IL-8* and *LITAF* mRNA levels were either significantly down-regulated or unchanged. Relative mRNA expression was determined by real-time PCR normalised to 18S rRNA. Data points are the mean of three biological replicates with error bars as standard deviation (*p < 0.05).

Messenger RNA expression levels of *LITAF*, *IL-6* and *IL-8* were significantly up-regulated (*p* < 0.05) in chicken cells infected with LPAI-H2N3 (Figure 4A), H5N1-tyEng91 (Figure 4C) or H5N1-tyTR05 viruses (Figure 4E). However, higher fold increase in the expression was observed in HPAI viral infections (Figures 4C and E) compared with LPAI virus infection (Figure 4A) in chicken cells. In contrast, the three pro-inflammatory genes were either significantly down-regulated (*p* < 0.05) or not significantly altered (*p* > 0.05) in duck cells infected with LPAI H2N3 (Figure 4B), H5N1-tyEng91 (Figure 4D) or H5N1-tyTR05 viruses (Figure 4 F).

**Figure 5:**
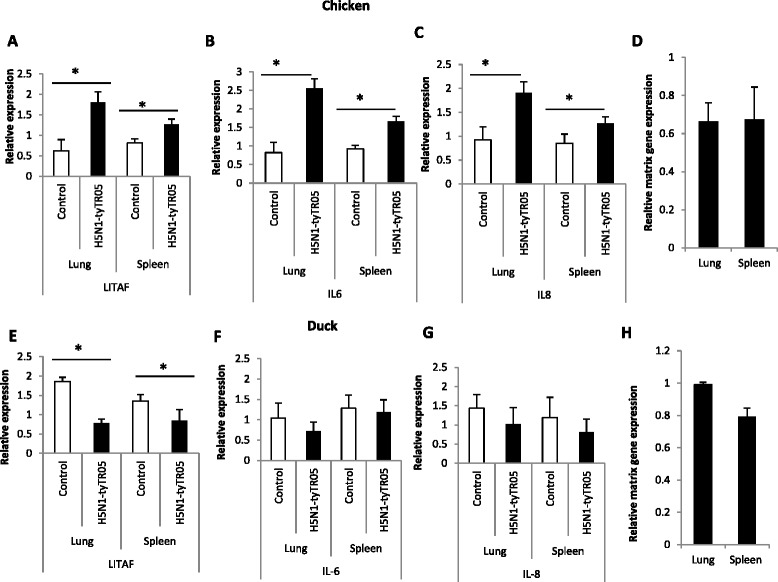
**Pro-inflammatory cytokine gene response to H5N1 virus challenge in chickens and ducks.** In the lungs and spleens of 3-weeks-old chickens at 24 h following infection with H5N1-tyTR05 virus, mRNA expression levels of **(A)** *LITAF*, **(B)** *IL-6* and **(C)** *IL-8* were significantly up-regulated compared with mock-infected controls. **(D)** Increased pro-inflammatory gene response in virus infected lungs correlated with RNA accumulation of influenza virus M-gene. In contrast, in the lungs and spleens of 4- weeks- old ducks infected with H5N1-tyTR05 virus, **(H)** despite viral M-gene RNA detection, **(E)** *LITAF* mRNA expression was significantly down-regulated and expression of **(F)** *IL-6* and **(G)** *IL-8* unaffected in relation to mock-infected controls. Relative mRNA expression was determined by real-time PCR normalised to 18S rRNA. Data points are the mean of three biological replicates with error bars as standard deviation.

Significant up-regulation (*p* < 0.05) of *LITAF* (Figure 5A), *IL-6* (Figure 5B) and *IL-8* (Figure 5C) was also detected in lung and spleen tissues from three-week-old chickens at 24 h of infection with H5N1-tyTR05 or H5N1-tyEng91 HPAI viruses (data not shown), along with abundance of virus matrix gene expression (Figure 5D). In lung and spleen tissues of 3-week-old Pekin ducks taken at 24 h of infection with H5N1-tyTR05 virus, despite the detection of virus matrix gene expression (Figure 5H), *LITAF* (Figure 5E) expression was significantly down-regulated (*p* < 0.05); *IL-6* (Figure 5F) and *IL-8* (Figure 5G) expression was not significantly affected (*p* > 0.05). In summary, similar elevated pro-inflammatory response in chickens but subdued pro-inflammatory response in ducks was observed in vitro and in vivo.

### Comparable type I Interferon response between influenza virus infected chickens and ducks

**Figure 6:**
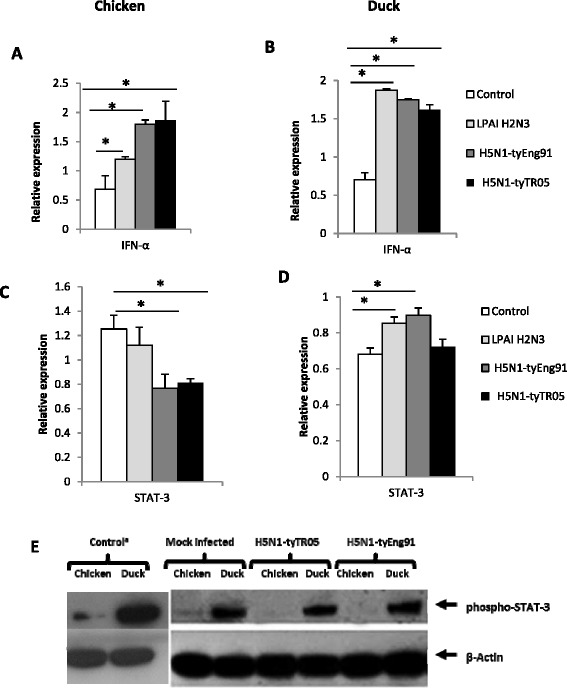
**Infected chicken and duck cells showed differential regulation of** ***STAT-3*** **.** *IFN-α* expression was significantly up-regulated in chicken **(A)** and duck **(B)** cells at 24 h following infection with LPAI-H2N3, H5N1-tyEng91 or H5N1-tyTR05 viruses. **(C)** While *STAT-3* expression in chicken cells was not significantly affected by LPAI-H2N3 virus infection it was significantly down-regulated in H5N1-tyEng91 and H5N1-tyTR05 virus infections. **(D)** In contrast *STAT-3* expression in duck cells was significantly up-regulated by LPAI-H2N3 or H5N1-tyEng91 viruses but was not affected by H5N1-tyTR05 virus infection. Relative mRNA expression was determined by real-time PCR normalised to 18S rRNA. Data points are the mean of three biological replicates with error bars as standard deviation (*p < 0.05). **(E)** Strong constitutive phospho-STAT-3 protein expression was detected in duck cells which was unaffected at 24 h following infection with H5N1-tyEng91 and H5N1-tyTR05 viruses. In chicken mock-infected cells, phospho-STAT3 protein expression was scarcely detectable and remained absent at 24 h following virus infection. ^a^Longer (5 min) exposure showing pSTAT-3 in chicken cells.

**Figure 7:**
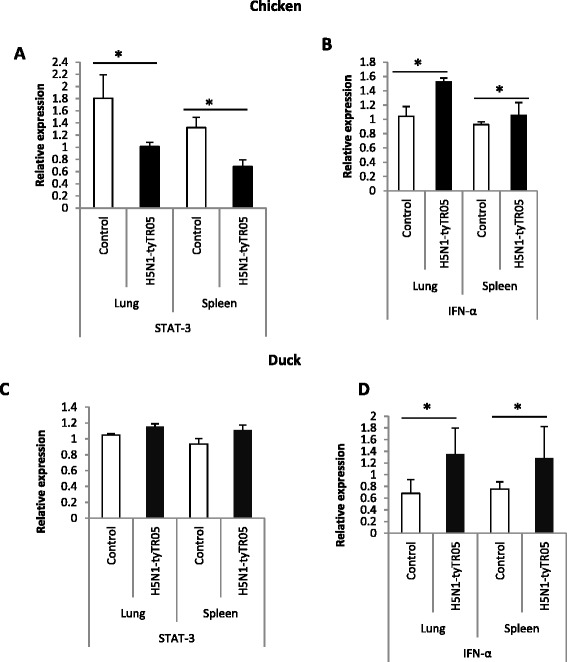
**Differential** ***STAT-3*** **regulation between H5N1 virus-infected chickens and ducks.** In the lungs and spleens of 3-weeks-old chickens at 24 h following infection with H5N1-tyTR05 viruses, **(A)** expression of *STAT-3* was significantly down-regulated whereas **(B)** *IFN-α* was significantly up-regulated. In the lung and spleen tissues from 4- weeks- old ducks at 24 h following infection with H5N1-tyTR05 virus, **(C)** *STAT-3* expression was unaffected and **(D)** *IFN-α* expression was significantly up-regulated. Relative mRNA expression was determined by real-time PCR normalised to 18S rRNA. Data points are the mean of three biological replicates with error bars as standard deviation (*p < 0.05).

Interferon alpha (*IFN-α*) expression was up-regulated in chicken (Figure 6A) and duck (Figure 6B) cells at 24 h of infection with LPAI-H2N3, H5N1-tyEng91 or H5N1-tyTR05 virus. Similar significant up-regulation of *IFN-α* expression was observed in the lung and spleen tissues of chickens at 24 h post-challenge with H5N1-tyTR05 (Figure 7B) or H5N1-tyEng91 HPAI viruses (data not shown). Up-regulation of *IFN-α* was also found in the lung and spleen tissues of ducks 24 h post-challenge with H5N1-tyTR05 HPAI virus (Figure 7D).

### Differential regulation of key components of JAK-STAT pathway between influenza virus infected chicken and duck cells

Contrasting transcriptional regulation of JAK-STAT pathway between infected chicken and duck cells was observed by microarray (Table 3). Following 24 h of infection with H5N1-tyEng91 or H5N1-tyTR05 virus, members of JAK-STAT signalling pathway (*JAK1*, *IFN-α receptor 1* [*IFNAR1*], *STAT-3* and *protein inhibitor of activated STAT 2* [*PIAS2*]) were down-regulated in chicken cells. In duck cells, expression of *IFNAR1*, *PIAS2* and *STAT-3* was up-regulated by LPAI H2N3 or H5N1-tyEng91 virus infection. Duck *JAK1* gene was removed during the probe masking procedure and hence its expression was not determined. Notably, in duck cells infected with H5N1-tyTR05 virus, *PIAS2* expression was down-regulated*, STAT3* was unchanged and *IFNAR1* was up-regulated.

*STAT-3* mRNA expression was validated in chicken and duck cells by qRT-PCR using the same total RNA samples that were used for microarray analysis. While *STAT-3* expression was not significantly affected (*P* > 0.05) by LPAI H2N3 virus infection, its expression was significantly reduced to half as much (*P* < 0.05) by H5N1-tyEng91 or H5N1-tyTR05 virus in infected chicken cells (Figure 6C). In duck cells, by contrast, *STAT-3* expression was significantly up-regulated (*P* < 0.05) by LPAI H2N3 and H5N1-tyEng91 virus infection; *STAT-3* expression was not significantly affected (*P* > 0.05) by H5N1-tyTR05 virus infection (Figure 6D). Similarly, *STAT-3* mRNA expression was down-regulated in the lung and spleen tissues of chickens challenged with H5N1-tyTR05 virus (Figure 7A) or H5N1-tyEng91 virus (data not shown).STAT-3 mRNA expression in the lung and spleen tissues was not significantly different (*p* > 0.05) in ducks challenged with H5N1-tyTR05 HPAI virus compared with controls (Figure 7C). In summary HPAI H5N1 virus infection resulted in down-regulation of key members of the JAK-STAT signalling pathway in chicken cells but not duck cells.

### STAT-3 appears to negatively regulate virus-induced pro-inflammatory response and promote virus replication in chicken and duck cells

We examined the phospho-STAT-3 protein expression in chicken and duck cells using a monoclonal antibody that is specific to pSTAT-3 at tyrosine-705. Phospho-STAT-3 protein in primary duck cells was expressed constitutively and remained strongly expressed at 24 h of infection with H5N1-tyEng91 or H5N1-tyTR05 virus (Figure 6E). In primary chicken cells, by contrast, phospho-STAT-3 was weakly expressed before infection and undetectable at 24 hpi with H5N1-tyEng91 or H5N1-tyTR05 virus (Figure 6E).

**Figure 8:**
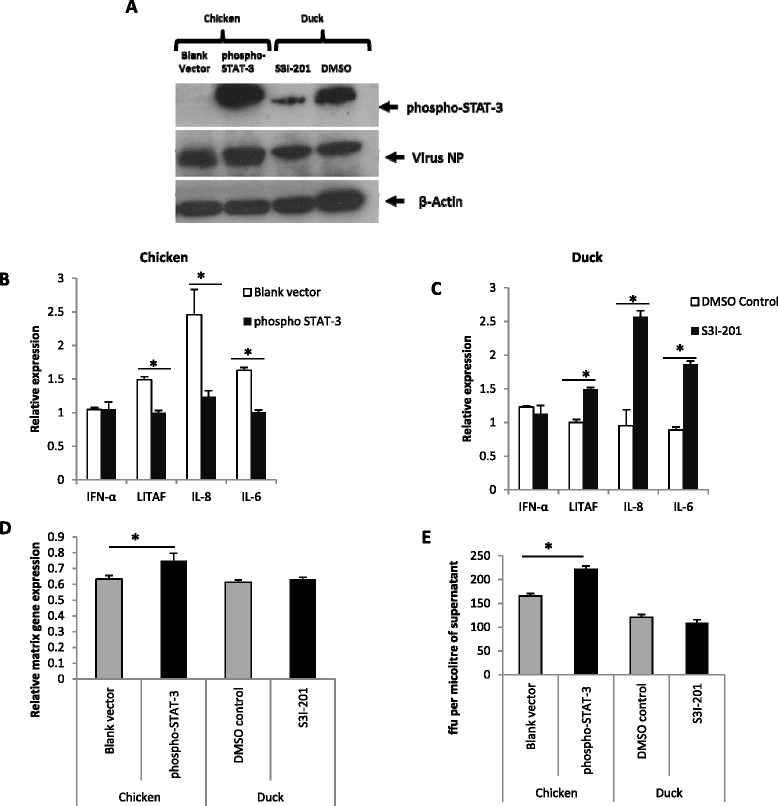
**STAT-3 appears to regulate the pro-inflammatory response and promote virus replication in H5N1 virus infected chicken and duck cells. (A)** Primary chicken embryo cells over-expressing phospho-STAT-3 showed a high phospho-STAT-3 expression while STAT-3 inhibitor S3I-201 treatment resulted in reduced phospho-STAT-3 protein expression in duck cells at 24 h following H5N1-tyEng91 virus infection (1.0 MOI). **(B)** phospho-STAT-3 over-expressing chicken cells showed a significant reduction in *LITAF*, *IL6* and *IL-8 mRNA* expression with no significant change in *IFN-α* expression. **(C)** At 24 h following H5N1-tyEng91 virus infection, in STAT-3 inhibited duck primary embryo cells, significant increase of LITAF, *IL-8* and *IL-6* mRNA expression was detected with no significant change in *IFN-α* expression. Phospho STAT-3 over-expression in chicken cells increased viral replication at 24 h following H5N1-tyEng91 virus infection as evidenced by increased detection of virus NP **(A)**, matrix gene mRNA **(D)** and infectious virus output in culture supernatant **(E)**. STAT-3 inhibition did not significantly affect virus NP **(A)** matrix gene expression **(D)** or infectious virus production **(E)** at 24 h following H5N1-tyEng91 virus infection in duck cells. Relative mRNA expression was determined by real-time PCR to 18S rRNA. Data points are the mean of three biological replicates with error bars as standard deviation (*p < 0.05).

To demonstrate a possible functional role of phospho-STAT-3 in mediating host pro-inflammatory response during influenza virus infection, primary chicken cells were transiently transfected with a phospho-STAT-3 expression plasmid, and duck cells were treated with STAT3 Inhibitor VI (S3I-201) prior to challenge with the H5N1-tyEng91 virus. High expression of p-STAT-3 protein in chicken cells over-expressing pSTAT-3 and reduced p-STAT-3 expression in duck cells treated with S3I-201 at 24 h following virus infection was detected by western blotting (Figure 8A).

Chicken cells transiently transfected with phospho-STAT-3 showed a significant (*p* < 0.05) reduction in *LITAF*, *IL-6* and *IL-8* mRNA expression following 24 h of H5N1-tyEng91 virus infection (Figure 8B). In duck cells treated with S3I-201, a significant (*p* < 0.05) increase of *LITAF*, *IL-6* and *IL-8* mRNA expression was observed at 24 h post H5N1-tyEng91 virus infection (Figure 8C). STAT-3 over expression in chicken cells or inhibition in duck cells had no significant (*p* > 0.05) effect on the expression of *IFN-α* expression following H5N1-tyEng91 virus infection. Chicken cells over- expressing phospho STAT-3 showed marginal increase in viral nucleo-protein (NP) expression (Figure 8A), significantly increased (*p* < 0.05) matrix gene mRNA expression (Figure 8D) and infectious virus release in culture supernatant (Figure 8E) at 24 h post H5N1-tyEng91 virus infection. STAT-3 inhibition had no effect on virus NP (Figure 8A), matrix gene expression (*p* > 0.05) (Figure 8D) or infectious virus production at 24 h post H5N1-tyEng91 virus infection in duck cells. In summary STAT-3 over-expression in chicken cells resulted in significant reduction whereas chemical inhibition of STAT-3 in duck cells resulted in significant increase in the proinflammatory gene response to H5N1 virus infection.

## Discussion

Microarray global gene expression analysis is a useful tool to gain important insights into the effects of influenza virus infection on host gene expression that could contribute to influenza pathogenesis [31]. As commercial high density microarray platforms are not yet available for many avian species, cross species hybridization using chicken oligonucleotide microarray is a useful tool to investigate gene expression in a range of avian species [32]. In this study, we successfully demonstrated that the Chicken GeneChip array could be used for the analysis of duck transcriptome as in the previous studies with woodchuck RNA on human microarrays [33] and pig RNA on human nylon microarrays [31]. However, direct high-throughput sequencing approach (RNA-Seq) is increasingly becoming popular. RNA-seq approach provides considerable advantages for examining transcriptome fine structure such as detection of allele-specific expression and splice junctions [34]. However, microarrays remain useful and accurate tools for measuring gene expression levels especially for cross-species studies where the full genome sequence and/or annotation are not available.

We found that influenza virus infection caused differential regulation of a greater number of genes involved in key biological functions in chicken cells compared with that in duck cells. Such changes in vivo could well account for the alterations in the function of infected cells and the pathogenesis of influenza virus in chicken. The relatively fewer changes in differential gene expression in infected duck cells suggest that cellular function was affected to a lesser degree than in chickens. HPAI viruses like H5N1 cause severe clinical disease in chickens and cause differential regulation of many genes involved in protein metabolism, translation, transcription, host defence/immune response, ubiquitination and the cell cycle [35].

Lethal influenza virus infections have been previously shown to cause an aberrant host innate immune response [18,36]. In this study we showed that HPAI virus infection caused an elevated immune gene response in chicken cells but not in duck cells. We previously showed that a moderated pro-inflammatory response plays an important role in mediating innate host resistance of pigs to H5N1 virus infection [17]. The present study found an elevated pro-inflammatory gene response in infected chickens and an attenuated inflammatory response in ducks following 24 h of infection with HPAI virus. The unusual severity of clinical human cases of H5N1 HPAI virus infection has been suggested to be linked to the hyperacute dysregulation of pro-inflammatory cytokines often referred as cytokine storm [14,15,37]. Tumour Necrosis Factor alpha (*TNF-α*) plays a major role in the development of clinical signs like fever and contributes to the lung lesions in humans [38] and pigs [39] during influenza virus infections. Due to absence of a “conventional” *TNF-α* gene in birds, expression of a lipopolysaccharide induced TNF alpha factor (*LITAF*) was analyzed in this study. LITAF gene has been previously shown to be very highly up regulated along with other pro-inflammatory cytokines following experimental inoculation of *E.coli* and *Salmonella* endotoxins [40] and LPAIV [41,42]. It is likely that the downstream pathways activated by *LITAF* might have a similar function as *TNF-α* in other species. We found an increased expression of *LITAF* in chickens and a down-regulated *LITAF* expression in ducks infected with H5N1-tyEng91 or H5N1-tyTR05 viruses. Similar increased expression of other pro-inflammatory cytokines *IL-6* and *IL-8* was observed in chickens, but not in ducks, infected with HPAIV [43].

However, pro-inflammatory response in ducks to different H5N1 virus strains could be inherently different. For example, a number of innate immune genes are up-regulated in the lungs of duck infected with a HPAI H5N1 virus (A/duck/Hubei/49/05), a LPAI H5N1 virus (A/goose/Hubei/65/05) [44] and a HPAI H5N1 virus (A/Vietnam/1203/04) [45]. In theory, the lack of such robust innate immune activation in ducks in vitro and in vivo following H5N1-tyTR05 virus could be due to inability of virus replication, or inherent differences in ducks response to different viral strains. However, H5N1-tyTR05 virus replicates to high titres in ducks with detectable viral shedding in oropharyngeal and cloacal swabs [12] and in vitro as shown by NP staining and M gene quantification. Hence, it is likely that there are inherent differences in the response of ducks to different subtypes of HPAI H5N1 viruses.

A previous study suggested that relative susceptibility of chickens to influenza virus, compared with ducks, could be due to the absence of RIG-I in chickens, a cytoplasmic RNA sensor that plays a key role in IFN mediated anti-viral responses [46]. However, a reduced IFN*-β* response in chicken cells in comparison with duck cells does not always seem to be a consistent observation in all influenza virus infections. Chicken peripheral blood mononuclear cells (PBMC) showed up-regulation of IFN*-β* while the levels of IFN*-β* are unaffected in duck PBMCs infected with a low pathogenic LPAI H11N9 influenza virus [47]. Type I interferon (IFN) response to AIV infection in chicken cells is mediated through melanoma differentiation-associated protein 5 (MDA-5) which chicken use to sense IAV infection [48]. Role of host IFN-α/β response in regulating virus replication is complex. In mice IFN-α/β causes either suppression or enhancement of hepatitis B virus (HBV) replication depending on the viral load [49]. Furthermore, a high host interferon IFN response to H5N1 HPAI virus may not, by itself, be sufficient to prevent a severe disease outcome. Conversely, host immune responses to HPAI H5N1 virus infection may contribute to disease pathogenesis. In human cases of HPAI H5N1 virus infection, higher levels of cytokines and chemokines were found in the blood of patients who died than those who survived [50-52].

The present study did not find any difference in IFN-α expression in chickens and ducks following H5N1 HPAI virus infection both in vitro and in vivo, raising a possibility that an IFN- α response by itself may not be sufficient to protect the host against virulent influenza virus infection. A study found strong up-regulation of IFN-γ mRNA in the lung and bursa of ducks but not chicken following infection with a LPAI H7N1 virus [53]. It is possible that IFN-γ rather than IFN- α or β could be important in protection against virulent influenza infection in avian hosts which warrants further studies.

In summary, we showed that host pro-inflammatory responses could be a key contributing factor to the pathogenesis of H5N1 influenza viruses and that the fatal outcome of H5N1 HPAI virus infection in chickens could be mediated by hyper-acute dysregulation of pro-inflammatory cytokines or the cytokine storm similar to human H5N1 HPAI virus infections. Furthermore, ducks showed attenuated pro-inflammation following infection with both the H5N1 viruses used in this study. However, to evaluate virus subtype specific differences, further comparative studies are required to assess the differences in cytokine response between ducks infected with different H5N1 virus isolates.

We found that *IL-18* was up-regulated in duck cells, but was down regulated in chicken cells infected with HPAIV. *IL-18* is involved in the control of influenza virus replication in the lungs of infected mice, especially at an early stage of infection, through activation of the innate immune mechanisms such as IFN and natural killer (NK) cells [54] and improves the early defence system by augmenting NK cell-mediated cytotoxicity. *IL-18* plays a critical role in the development of protective immunity against various intracellular pathogens including *Mycobacterium tuberculosis*, *Yersinia enterocolitica*, *Cryptococcus neoformans* and herpes simplex virus [55-58]. Recent studies demonstrated that recombinant vaccines simultaneously expressing influenza antigens along with IL-18 significantly enhance the protective efficacy of influenza vaccines in chicken [9]. A study also found that LPAI but not HPAI infection is associated with enhanced NK cell response in lungs of chicken [59], suggesting a crucial role of NK cell response in influenza virus pathogenesis. This evidence warrants further functional studies to investigate the mechanisms underlying the protective role of IL-18 and NK cell response during influenza virus infections in chicken.

The JAK-STAT signalling pathway is activated by the type I (IFN-α and IFN-β) and type II (IFN-γ) interferons [60] and is critical for a successful IFN-α antiviral response against virus infections [61]. This study found that key genes in JAK-STAT signalling pathway were down-regulated in chicken cells but were either up-regulated or unchanged in duck cells at 24 h following HPAIV infection. STAT-3, a key constituent of this pathway plays a critical role in the IFN signalling pathways and is required for a robust IFN-induced antiviral response [62].

STAT family proteins are activated by phosphorylation by JAKs on a single tyrosine in the C- terminus at position 705 that enables their homo- or hetero-dimerization. Dimerized STAT proteins subsequently migrate to the nucleus and stimulate transcription [63]. Hence phosphorylation of STAT-3 at tyrosine- 705 is a key indicator of its DNA binding ability and activity as a transcription factor. We found that duck cells had a high basal expression of pSTAT-3 (Tyr705) compared with chicken cells. pSTAT-3 (Tyr705) protein expression was undetectable in chicken cells 24 h after infection with HPAI H5N1 virus while it was unaffected during infection of duck cells. We could not verify STAT-3 protein expression in-vivo due to unavailability of protein samples from HPAI infected chickens and ducks. The transcriptional down-regulation of *STAT-3* with corresponding lack of pSTAT-3 protein expression in chicken cells suggests that H5N1 HPAI virus infection inhibits STAT-3 mediated gene transcription and/or activation. An important function of STAT-3 is its antagonistic effect on the inflammatory response. Activation of the STAT-3 signaling pathway promotes a strong anti-inflammatory response thereby blocking the inflammatory cytokine response [64]. Hence, it is likely that the excessive pro-inflammatory response in H5N1 HPAI virus infected chickens could be mediated through the inhibition of STAT-3 and a functional STAT-3 corresponds to an attenuated pro-inflammation in H5N1 virus infected ducks. In addition, *IL-18* stimulation results in enhanced tyrosine phosphorylation of STAT-3 [65]. In the present study *IL-18* activation correlated with increased STAT-3 phophorylation in duck cells while down-regulation of *IL-18* in H5N1virus infected chicken cells correlated with reduced pSTAT-3 detection.

Chicken cells over-expressing constitutively active STAT-3 showed significantly lower *LITAF, IL-6* and *IL-8* mRNA levels compared with the blank plasmid transfected cells at 24 h post H5N1-tyEng91 virus infection. Duck cells treated with S3I-201 that inhibits the transcriptional activity of STAT-3, resulted in increased expression of *LITAF, IL-6* and *IL-8* compared with control cells at 24 h post H5N1-tyEng91 virus infection. Previous studies showed that constitutively active STAT3 can suppress both *IL-6* and *TNF-α* production in lipopolysaccharide-stimulated macrophages [66]. The sum of this evidence raise a strong possibility that STAT-3 mediated gene transcription could play a central role in suppressing pro-inflammatory responses during H5N1 virus infection in ducks. Furthermore the ability of HPAI viruses to inhibit STAT-3 in chickens correlates with excessive pro-inflammatory response and the development of fatal disease. Further studies would help to identify candidate genes that suppress pro-inflammation during HPAI H5N1 virus infection and are transcriptionally regulated by STAT-3.

Surprisingly, we found that STAT-3 over-expression significantly increased H5N1 HPAI virus replication in chicken cells while STAT-3 inhibition had no significant effect on virus replication in duck cells. The increase in influenza virus replication in chicken cells over-expressing STAT-3 could be due to inhibition of type I IFN-mediated antiviral response. However, STAT-3 over-expression or inhibition did not significantly affect *IFN-α* mRNA expression in chicken and duck cells respectively. Conversely, STAT3 has been suggested as an important upstream element in type I IFN signal transduction and in the induction of antiviral activities [62]. The role of STAT-3 in virus replication appears to be complex. For example STAT-3 induction promotes varicella-zoster virus replication [67], activates anti-hepatitis C virus (HCV) activity in liver cells [68] and promotes HCV RNA replication [69]. Findings of the present study suggest that STAT-3 could promote influenza virus replication in chicken cells but not in duck cells. We showed previously that duck cells produce significantly less infectious influenza virus compared with chicken cells which correlated with rapid cell death [20]. It is likely that other mechanisms independent of STAT-3 could contribute to the antiviral effects observed in duck cells. As STAT-3 is a transcription factor and known to mediate the expression of a variety of genes, it is likely that STAT-3 over-expression or inhibition may affect a number of cellular signalling pathways. Hence, further studies are needed to identify candidate genes that play an important role in mediating pro-inflammation and influenza virus replication in chickens and ducks.

## Additional files

Additional file 1:
**Preparation of RNA target and hybridization on to GeneChip expression arrays.** Protocol describing sample preparation and hybridization on to Genechip and probe masking for cross species hybridization of duck RNA onto chicken Genechip. Additional file 2:
**List of key immune related genes that were differentially regulated between chicken and duck cells following influenza virus infection.** List of key immune related genes that were differentially regulated in chicken and duck cells following infection with LPAI H2N3, H5N1-tyEng91 and H5N1-tyTR05 viruses.
