# Supplementary material for: Highly pathogenic avian influenza virus infection in chickens but not ducks is associated with elevated host immune and pro-inflammatory responses
Source: Vet Res. 2014 Nov 28;45(1):118. doi: 10.1186/s13567-014-0118-3 (PMC4246556; doi:10.1186/s13567-014-0118-3)
Supplement: Additional file 1: — Preparation of RNA target and hybridization on to GeneChip expression arrays. Protocol describing sample preparation and hybridization on to Genechip and probe masking for cross species hybridization of duck RNA onto chicken Genechip. [file 13567_2014_118_MOESM1_ESM.doc]

**Preparation of RNA target and hybridization on to GeneChip expression arrays**

Each RNA sample was hybridized on to one GeneChip chicken genome array (Affymetrix) and a total of 16 array chips were used in the study.

RNA targets for labelling were prepared using the GeneChip® 3′ IVT Express Kit (Affymetrix) following the manufacturer’s instructions. Briefly, the total RNA was reverse transcribed to synthesize first-strand cDNA, which was then converted into a double-stranded DNA template for transcription. Anti-sense amplified RNA (aRNA) was synthesized by in-vitro transcription incorporating a biotin-conjugated nucleotide. The aRNA was then purified to remove unincorporated NTPs, salts, enzymes, and inorganic phosphate. The biotin-labelled aRNA was then fragmented prior to hybridization onto GeneChip 3′ expression arrays. Hybridization of labeled target on to GeneChip probe arrays was carried out using GeneChip Hybridization, Wash, and Stain Kit (Affymetrix) following the manufacturer’s instructions. After 16hrs hybridization at 45 °C, probe arrays were removed from the oven and the hybridization cocktail was extracted with a micropipette. Washing and staining of probe arrays was carried out using GeneChipHybridization Wash and Stain Kit and GeneChipFluidics Station 450 (Affymetrix) following the manufacturer’s instructions. Probe arrays were then scanned using GeneChip Scanner 3000 with AGCC scan control software (Affymetrix) to generate probe intensity data (.cel file) for each of the arrays.

### Microarray data analysis

Microarray expression data was analyzed using the GeneSpring GX11 expression analysis software (Agilent Technologies). Using the Advanced Workflow option, probe summarization was carried out by Robust Multichip Averaging (RMA) summarization algorithm [63,64], then baseline transformation was carried out by baseline to median of all samples. Arrays from virus and mock infected samples were grouped, based on treatment, with two replicate arrays in each group. Statistical analysis was carried out by analysis of variance (ANOVA) with a *p* value cut-off of 0.05 by asymptotic p-value computation algorithm with no multiple testing correction. The entities satisfying the significance analysis were passed on for the fold change analysis. *Fold Change Analysis* was used to identify genes with expression ratios of treatment and control samples that exceeded a cut-off of 1.3. All the genes that were significantly regulated by a fold change difference of ± 1.3 (*p* < 0.05) were grouped into gene ontology (GO) terms using the *GO analysis* function in GeneSpring software. Genes were grouped into cellular component, biological process and molecular function GO terms. The percentage of differentially regulated genes in each of these GO terms was determined from the gene expression profiles for each of the virus infected compared with the mock infected chicken and duck cells. Differentially regulated genes after each virus treatment were grouped into functional clusters using DAVID bioinformatics resources version 6.7 [65,66].

### Cross species array analysis

### *Probe masking*

### A previously described genomic DNA (gDNA) based probe-selection method [67] was used to maximise the sensitivity of the chicken GeneChip to study the transcriptome of duck. Briefly genomic DNA from Pekin duck (*Anas platyrhynchos*) primary lung cells was biotin-labelled and hybridized to the Chicken (*Gallus gallus*) GeneChip array and a probe intensity data file (*cel*) was generated as described above. Probe-sets on the chicken chip were selected for subsequent duck transcriptome analyses if the probe-set was represented by perfect match (PM) probes with duck gDNA hybridization intensities above an experimentally set threshold. Selection was performed using a *cel* file parser script written in the Perl programming language, X-species Version 2.1 [68]. After installing Active Perl software version 5.10.1.1007 for windows, *CDF_masking.zip* was downloaded and unzipped to a chosen location on the computer [69]. Original CDF file for chicken chip downloaded from Affymetrix website and duck gDNA hybridization *cel* file were copied to CDF-masking folder. In the CDF-masking folder *easy_script.pl* was run to generate a series of probe mask (CDF) files for duck with a range of threshold values. Using this method, 20 probe mask files were generated with gDNA hybridization intensity thresholds ranging from 20 to 2000.

### *Analysis of cross species hybridization data*

### Perfect match (PM) probes of chicken genome array hybridized extensively to the *Anas platyrhynchos* genomic DNA. When the gDNA hybridization intensity threshold was increased from 20 to 2000, probe pair retention in the probe mask files decreased rapidly. However, the retention of whole probe-sets, representing transcripts, was less sensitive to the increase in gDNA hybridization intensities during probe mask file generation. This is because only a minimum of one probe pair is required to retain a probe-set. For example probe mask file generated using a gDNA hybridization intensity threshold of 20 retained 100% *Gallus gallus* probe-pairs and probe-sets (i.e. 423 199 and 38 473 respectively). Whereas the probe mask file generated with a gDNA intensity threshold of 100, masked over 50% of probe pairs, while only 2.5% of *Gallus gallus* probe-sets were masked (retaining 97.5% probe sets).

### Duck transcriptome analysis

### Using the create technology function in Genespring Gx11, a technology was created using the duck CDF file for each of the gDNA intensity thresholds tested. The probes were annotated using the chicken GeneChip annotation file. The *cel* files obtained by hybridizing each of the virus and mock infected duck total RNAs on to chicken GeneChip were analyzed as described above using the technology created for duck. A series of 11 analyses were performed for each gDNA intensity threshold from 40-450. Out of the 11 gDNA intensities analyzed, a threshold of 200 gave the highest number of genes that are significantly differentially regulated (*p* < 0.05), differentially regulated with a fold change of ± 2 and significantly differentially regulated with a fold change of ± 2 (*p*< 0.05). Based on these findings, technology created with a gDNA threshold of 200 was used for further transcriptome analysis of all the duck samples. Technology created for duck represented 32 896 transcripts out of the total 38 535 transcripts represented in the original chicken GeneChip technology.
